# Functional modelling of planar cell polarity: an approach for identifying molecular function

**DOI:** 10.1186/1471-213X-13-20

**Published:** 2013-05-14

**Authors:** Lee D Hazelwood, John M Hancock

**Affiliations:** 1 Faculty of Biological Sciences, University of Leeds, Leeds LS2 9JT, UK; 2 MRC Mammalian Genetics Unit, Harwell Science and Innovation Campus, Oxfordshire OX11 0RD, UK; 3 Department of Physiology, Development & Neuroscience, Cambridge University, Cambridge CB2 3EG, UK

**Keywords:** Planar polarity, PCP, Mathematical modelling, Self organisation, *Drosophila*, *In*-*silico* phenotyping

## Abstract

**Background:**

Cells in some tissues acquire a polarisation in the plane of the tissue in addition to apical-basal polarity. This polarisation is commonly known as planar cell polarity and has been found to be important in developmental processes, as planar polarity is required to define the in-plane tissue coordinate system at the cellular level.

**Results:**

We have built an *in*-*silico* functional model of cellular polarisation that includes cellular asymmetry, cell-cell signalling and a response to a global cue. The model has been validated and parameterised against domineering non-autonomous wing hair phenotypes in *Drosophila*.

**Conclusions:**

We have carried out a systematic comparison of *in*-*silico* polarity phenotypes with patterns observed *in vivo* under different genetic manipulations in the wing. This has allowed us to classify the specific functional roles of proteins involved in generating cell polarity, providing new hypotheses about their specific functions, in particular for Pk and Dsh. The predictions from the model allow direct assignment of functional roles of genes from genetic mosaic analysis of *Drosophila* wings.

## Background

Symmetry breaking in nature is fundamental to life and to many biological processes, occurring over a range of scales from the asymmetry of molecules through to large complex organs and the organism as a whole. Understanding how the asymmetry at a molecular level is mechanistically communicated through to the cellular, tissue and organ scales presents us with a formidable challenge.

Studies of gastrulation in *Xenopus*[1,2] show that development of complex tissues requires a series of well-choreographed cellular movements and shape changes to generate complex tissue shapes. One requirement for any general tissue shape change is the formation of a three dimensional coordinate system, the cartesian coordinate axes x,y and z, for example. In a sheet of cells, the first axis that forms is the z axis perpendicular to the sheet. This is associated with the appearance of apical-basal polarity. Once this axis is established, cells may then arrange themselves within the tissue plane and in doing so create the possibility of defining orthogonal x and y axes in the tissue plane. The associated in-plane polarity is referred to as planar polarity or planar cell polarity (PCP).

The fundamental nature of planar cell polarity means that its malfunction can have a major impact on an organism’s development. To date, PCP has been implicated in the correct development of many tissues, including the neural tube [3], lung [4], kidney [5,6] and it has also been implicated in cancer [7]. Unfortunately, these tissues are difficult to observe *in vivo* and they also lack easily visible polarity markers from which to build conceptual models.

**Figure 1:**
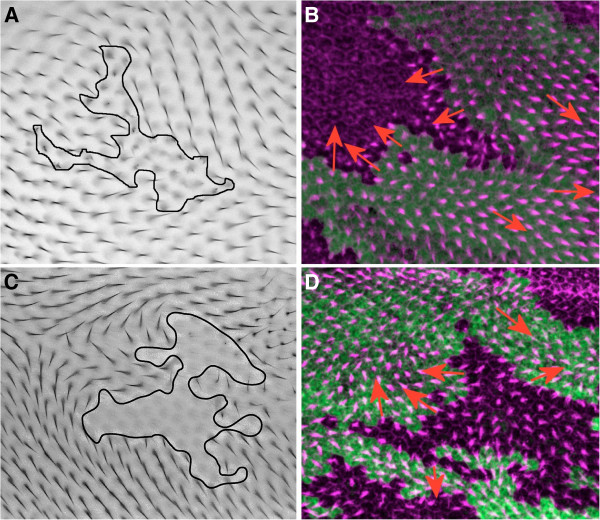
***Fz *and *Vang *clones in the adult and pupal wing.***fz* clones in an adult wing (**A**, courtesy of Paul Adler, unpublished) and the pupal wing (**B**, courtesy of David Strutt, unpublished) and *Vang* clones in the adult (**C**, republished with permission, Genetics Society of America, Genetics.org [11] and pupal wing (**D**, courtesy of David Strutt, unpublished). Clone outlines are shown in black (**A** and **C**) or purple in (**B** and **D**). Arrows indicate trichome direction in **B** and **D**. Notice that the presence of the clone influences the hairs to point toward the clone in **A**, which is more pronounced or locally normal for the case of pre-hairs in **B**. The presence of the clone influences the hairs to point away from the clone in **C**, which is more pronounced or locally normal for the case of pre-hairs in **D**.

*Drosophila*, and the *Drosophila* wing in particular, are much more experimentally tractable than mammalian systems and serve as a powerful experimental system for investigating the relationship between the molecular machinery required for coordinating local and global polarity. The majority of investigations in the *Drosophila* wing that aim to determine the mechanism of planar polarity have used genetic mosaic analysis, in which small groups of cells (“clones”) that lack the activity of a particular gene are generated. Clones of cells that lack activity of the proteins Fz or Vang produce interesting polarity patterns, known as domineering non-autonomy [8-11], imposing a prescribed polarisation on cells adjacent to the clone boundary. Examples of domineering non-autonomous patterns are shown in both the adult and pupal wing for *fz* clones (with hairs adopting an attractive pattern) and *Vang* clones (with hairs adopting a repulsive pattern) in Figure 1. This domineering patterning is in contrast to wildtype wings and mosaic clones of factors that act autonomously [12-14], where the cells organise themselves with common polarisation.

Interpretation of these studies has led to the identification of a “core” group of planar polarity proteins which have conserved functions in vertebrates [15], and serve the primary function of coordinating polarity between neighbouring cells [16]. The most important of these are the transmembrane proteins Frizzled (Fz), Flamingo (Fmi, also known as Starry Night) and Van Gogh (Vang, also known as Strabismus). These proteins couple to the apical-basal asymmetry by localising together in the adherens junction zones of cells. Here they mediate cell-cell interactions to establish the local in-plane cellular symmetry. This “core” pathway is also known to be mediated by three cytoplasmic proteins: Prickle (Pk), Dishevelled (Dsh) and Diego. In addition to the core pathway, studies in the *Drosophila* abdomen and larval epidermis indicate that in some contexts the cadherins Fat (Ft) and Dachsous (Ds) and the Golgi-localised protein Four-jointed (Fj) can act independently to establish planar polarity [17,18].

As well as local coordination between cells, polarisation on a global scale requires coupling to a non-local signal. This signal or cue that orientates or re-orientates polarity could arise from a morphogen gradient, a gradient of expression or activity of the core PCP proteins [19], or could be due to cellular movement [20], for example. There are two theoretical possibilities for how this might take place. The first is that the core PCP machinery can function independently of a global signal, with the global signal providing a guiding role. This is the basis of models presented for the roles of gradients of Fat (Ft) and Dachsous (Ds) [21-23]. The second possibility is that the global signal is intrinsic to core PCP protein function, acting to generate both local intercellular and long-range polarisation. This is the basis of models proposing a Fz gradient in global signalling [10,24-26]. Cellular movement could involve either or both possibilities [20,27]. In all cases, the precise molecular functions are still unclear, with alternative evidence to support different possibilities.

Experiments involving clones are particularly important for investigating the precise molecular roles of proteins as they create an environment where the local and global coordination machinery are potentially in conflict. This leads to the domineering non-autonomous phenotypes, where the hair orientation is locally nearly perpendicular to the clone boundary but relaxes to the wildtype tissue orientation over several cell diameters. The relative importance of molecular or physical factors that determine the local and global behaviour is difficult to assess without placing these factors within a quantitative mathematical model.

Mathematical modelling has played an increasingly important role in the validation of conceptual models of cell polarity [23,24]. Common to all cell polarity models is the presence of feedback loops, which are required to generate the asymmetric localisation of proteins that specify polarisation within the plane. Feedback within a system or a model can be created through the direct inhibition of reaction species or ligand binding, as is proposed in the case of Vang inhibition of Fz [28]. Alternatively, feedback can also be created by a preferential activation of one intercellular complex over another [20,29,30] and more specifically a preference of Fz-Fmi receptor binding over Vang-Fmi at a particular cell edge [25].

The principle drawback of the modelling approaches that have so far been applied to PCP is that by attempting to capture all the relevant biological and physical interactions model clarity is often compromised. This includes the introduction of many un-measurable parameters all of which create problems in their validation and interpretation. In this paper, we aim to show that modelling can be applied intuitively to wing hair phenotypes and used to infer the molecular function of proteins using genetic studies.

More specifically, the aims of this paper are to i) formally describe a cellular measure of polarisation, ii) build a functional model of planar cell polarity based on the concept of a cellular polarisation, iii) validate and parameterise the model using genetic clones that lead to domineering non-autonomous phenotypes in *Drosophila* wings, iv) carry out a systematic *in*-*silico* “knock-out” of the model parts and v) compare the experimentally observed hair polarity phenotypes with those generated *in*-*silico* to identify the molecular function of proteins.

**Figure 2:**
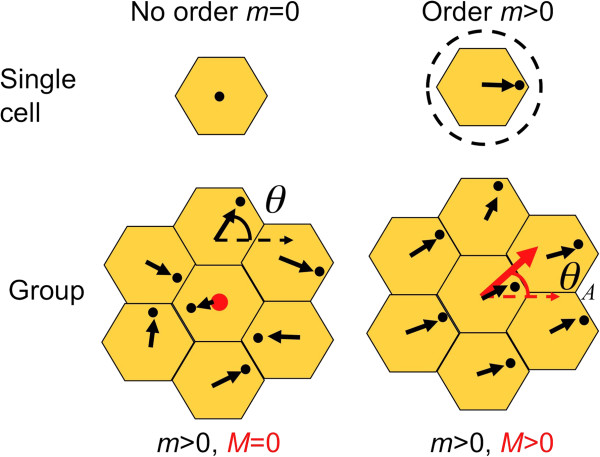
**Diagrammatic representation of single and group order parameters.** Top: Single cell order parameter with zero cell polarisation *m =* 0 (left) and finite polarisation *m* > 0 (right). Bottom: Group orientational order parameter with zero group polarisation *M =* 0, but with finite single cell order *m* > 0 (left). Finite group orientational order *M* > 0 and single cell order *m* > 0 (right). Single cell order parameter vector **m** and group orientational order parameter **M** are shown as black and red arrows respectively. θ is described for either a single cell orientation or the average orientation for a group of cells θ_A_, relative to distal.

**Figure 3:**
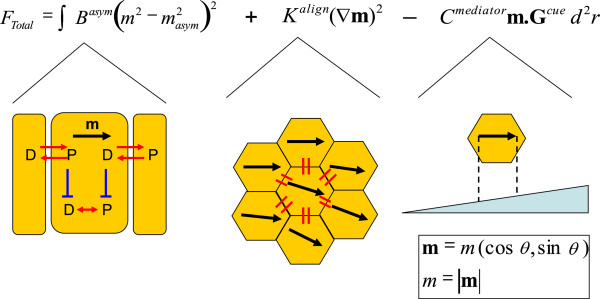
**Mathematical model of planar polarity.** The first term controls a cell’s polarisation, which in physical terms would relate to the underlying asymmetry in the proximal (P) and distal (D) proteins, whose strength is determined by *B*^*asym*^ and has a stable asymmetric state of magnitude *m*_*asym*_. The schematic beneath illustrates a possible simplistic feedback pathway. The second term enforces uniformly orientated polarisation with its resistance to distortion determined by *K*^*align*^ , and ultimately the inter-cellular interactions between P and D proteins. The schematic beneath illustrates the necessary cell-cell interactions. The third term linearly couples the cell’s polarisation **m** to an increasing cue **G**^cue^, with the response and mediation to that cue by *C*^*mediator*^. The schematic beneath illustrates an increasing global cue’s differential action on a cell. The equation has been written in terms of a polarity vector (term 2 and 3) or its magnitude (term 1).

To address these issues, we have built a functional model of planar polarity in which the detailed molecular interactions have been integrated into a polarity measure **m** at the cellular scale and **M** at the group scale. The model includes mathematical terms that account for the cell’s ability to maintain its own intracellular polarisation (parameter B), interact with the polarity of adjoining cells (parameter K) and interact with a global cue (parameters C and G), see Methods and Figures 2, 3 for details. In this way, we can step back from the detail and ask more general questions from our model in relation to the observed polarity phenotypes at the tissue scale. Our approach is based on the Ginzburg-Landau method used routinely to understanding ordering processes within condensed matter physics [31,32] and is similar to those used in the study of mouse hair patterning [33] and *Drosophila* hair swirling [29,34]. Our application of this physical or functional model is original in that we have clearly identified the biologically relevant components and used polarity patterns in the vicinity of genetic mosaics and “clones” to infer protein function.

We have chosen to model the local ordering properties in a flat monolayer of hexagonal cells, analogous to the situation found in the *Drosophila* wing. This model system has the unique advantage that the polarity is easily observed in terms of wing hair initiation or final hair orientation. We also restrict our focus to polarity patterns observed in the vicinity of clones on genetic backgrounds of the “core” planar polarity proteins (Fz, Vang, Fmi, Pk and Dsh) and those from the “Ft/Ds system” (Ft, Ds, Fj). This comparison will be primarily carried out using published images of pre-hair initiation sites whose location will be our polarity indicator. We believe these have advantages over adult hair polarity patterns in that pre-hair patterns are subject to fewer downstream effects and are more easily defined within our approach. Adult hair patterns are however included wherever possible. Protein expression patterns have not been included as they are more difficult to observe and interpret due to the technical difficulty of simultaneously imaging multiple proteins using fluorescent markers.

## Results

### Parameterisation

In order to parameterise our model it is important to remind ourselves what key features we are looking to reproduce in our simulations. The archetypal examples for attractive clones are a loss of *fz*[8,9] and for repulsive clones a loss of *Vang*[11], shown in Figure 1A and C respectively for adult wings and repeated in pupal wings in Figure 1B and D. The polarity adopts a near locally perpendicular orientation at the clone boundary, which re-orientates towards the global or far field orientation within several cell diameters. Quantitative measurements of this re-orientation do not currently exist in the literature, which focuses more on qualitative features. However, we have attempted to measure the range of values for re-orientation towards the global direction from published images. For pupal wings we found the relaxation, in the direction perpendicular to the proximodistal axis, took place over 3-5 cells [14,35] and for adult wings the relaxation was slightly longer, over 4-5 cells [11,26,36,37]. Angular measurements were subject to a less than 5 degree error. This has been incorporated into the cell range.

Fortunately, it is not necessary to find absolute parameter values for our model since equilibrium polarity patterns can be completely defined in terms of the ratios of parameters. Therefore we carried out a systematic exploration of parameter ratios that yield polarity patterns consistent with the archetypal *fz* and *Vang* clones. To ensure that the patterns were quantifiably similar to those observed we also measured the distance over which the orientation relaxes to the proximodistal direction and the distance over which the magnitude of polarity is close to wildtype. The resulting plots or phase diagrams can be found in Additional file 1: Figure S1 and Additional file 2: Figure S2. These plots calculate explicitly the length scales for re-orientation and polarisation generation, though we have attempted to represent them in a format accessible to biologists. These scales correspond to relaxation lengths in physics, whose derivation are beyond the scope of this paper.

**Figure 4:**
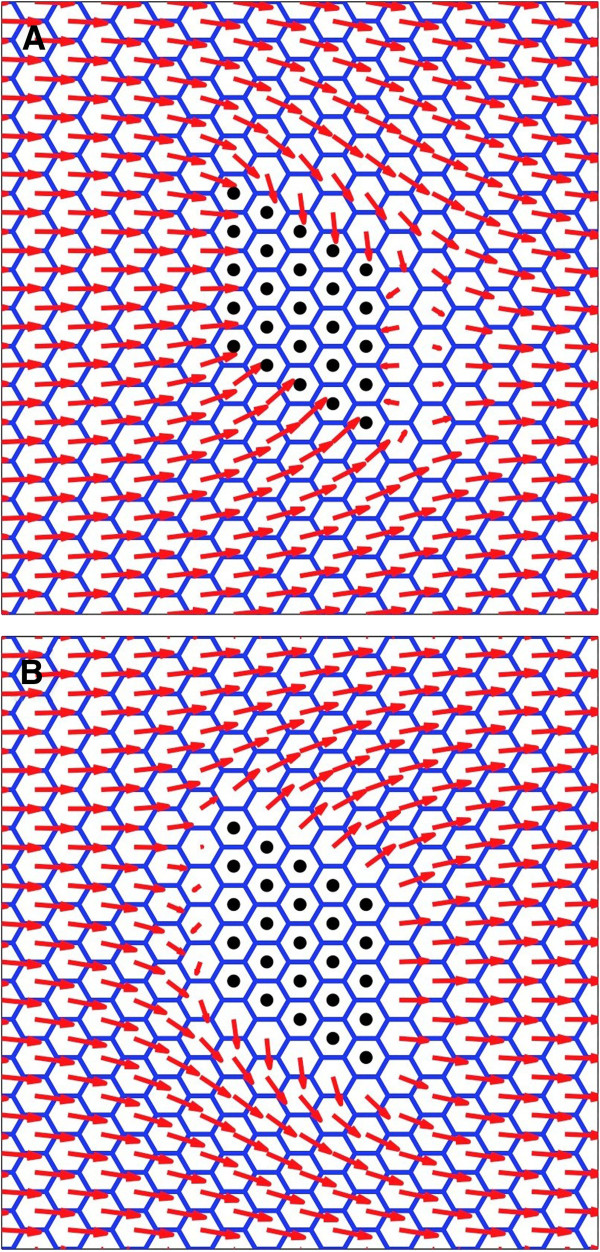
**Simulation of clones in a WT background.** (**A**) Attractive clone (*fz*) simulation. Polarisation vector is wildtype (proximal-distal) away from the clone and points inwards in the vicinity of the clone. (**B**) Repulsive (*Vang*) clone simulation. Polarisation vector is wildtype proximal-distal away from the clone and points outwards in the vicinity of the clone. Clone is the same 5×5 marked rhombus shaped region in each simulation.

Additional file 1: Figure S1 indicates that in order for patterns to exhibit a relaxation in the range of 3-5 cells then the parameter *K*^*align*^, maintaining polarity alignment between cells, must be greater than or equal to *B*^*asym*^, controlling the free energy costs of departures from the **intra**cellular cell polarisation *m*_*asym*_, i.e. the tendency of Fz and Vang not to colocalise within the cell. An additional constraint in determining a relaxation range of 3-5 cells is that the magnitude of the free energy required to maintain an individual cell’s polarisation *B*^*asym*^, is approximately ten times greater than that to couple the cell polarity to the global signal C^mediator^ G^cue^. For the purposes of our simulations we then choose the first point where both constraints were achieved i.e. *B*^*asym*^ = *K*^*align*^ and *B*^*asym*^*=* 0.1 × C^mediator^G^cue^, as indicated in Additional file 1: Figure S1. These parameters have been used to generate the *in-silico* attractive clone as shown in Figure 4A (compare to *fz* clone in Figure 1A & B) and a repulsive clone as shown in Figure 4B (compare to *Vang* clones in Figure 1C & D). Additional file 2: Figure S2 provides assurance that this choice of parameters also leads to cells acquiring a wildtype level of polarisation magnitude, though not necessarily wildtype direction.

### Systematic investigation of polarity phenotypes

For the three model terms, for an attractive clone there are 8 combinatorial possibilities for complete *in-silico* ‘functional knockout backgrounds’ of *m*_*asym*_, *K*^*align*^ and *C*^*mediator*^***G***^*cue*^ including one all wildtype and one all knockout. The wildtype background has already been presented in Figure 4 and the all knockout background is not interesting as there is no polarity anywhere. We did not vary B^asym^ as it is always non-zero due to the thermodynamic constraints discussed in Methods, though it could become important when comparing experiments carried out at different temperatures.

We therefore present both single and double *in-silico* background knockout possibilities and for attractive (*fz*) clones only. The repulsive (*Vang*) clones create the corresponding reversed polarity patterns and can be directly inferred by reversing the arrows of the attractive clones. Restricting ourselves to these two generic clones, and their associated patterns in the wildtype, is fundamental to our approach. It allows us to clearly infer functional roles induced by changing genetic backgrounds, assuming the clone boundary conditions remain unchanged.

#### Intrinsic polarity generation absent (model: m_asym_=0)

**Figure 5:**
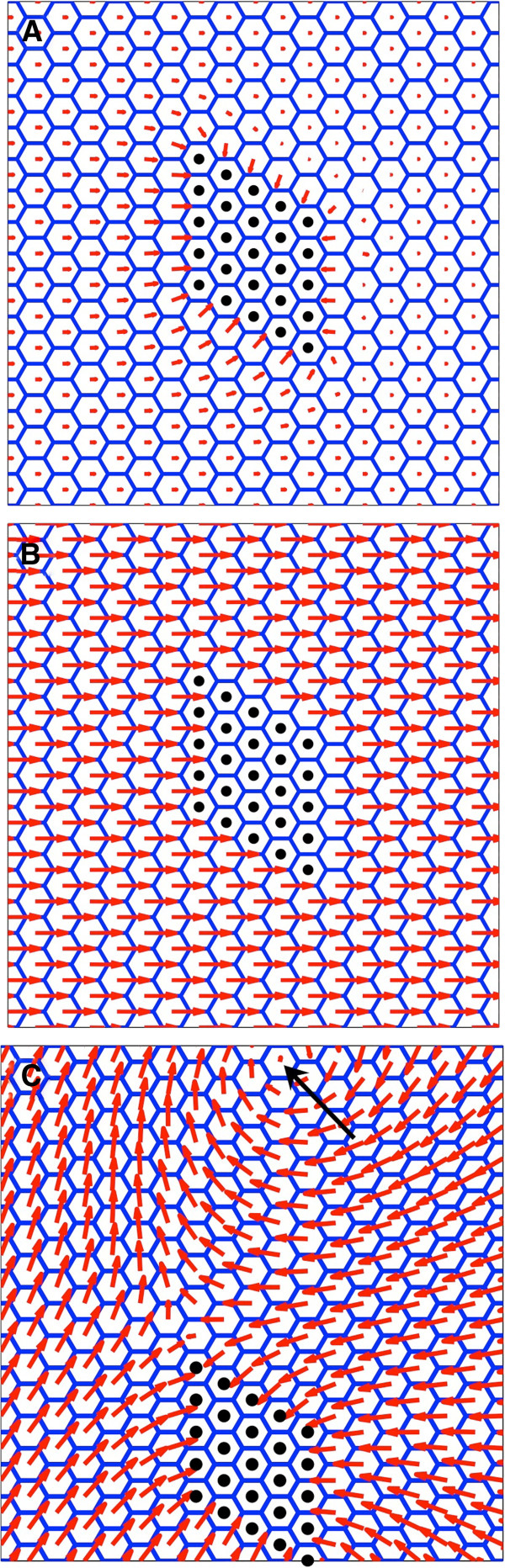
**Simulation of clones in a single mutant background.** Simulations of single background knockouts using attractive (*fz*) clone. (**A**) *m*_*asym*_*= 0* (asymmetrically driven cell polarity is zero). (**B**) *K*^*align*^*= 0* (intercellular polarity alignment forces absent). (**C**) *C*^*med*^***G***^*cue*^*= 0* (no globally orientating signal or a response to it). Clone has been shifted down in (**C**) to emphasise the vortex swirl, indicated by the arrow. Repulsive (*Vang*) clones can be inferred directly by reversing the arrows of the attractive (*fz*) clones.

The first single *in-silico* background knockout corresponds to each cell being no longer able to generate a naturally polarised state. Physically, this might be due to the polarity system being no longer able to self-organise proximal and distal proteins asymmetrically within the cell without an external influence. Figure 5A shows the *in-silico* polarity phenotype for an attractive (classic *fz*) clone on a background of cells where *m*_*asym*_*=* 0. The clone is seen to induce some polarisation on cells in the direct vicinity of the clone, though the effect is short ranged. The global signal in this case is insufficient to generate measurable polarisation away from the clone. The polarity vectors extend a shorter distance in the proximal direction, which is due to the opposition of the global signal. This leads to a reduction in polarity on the distal side of the clone, close to the clone boundary.

The only comparable experimental observation that is similar to this phenotype is the pre-hair polarity pattern seen with a *fz* overexpressing clone in a *dsh*^*1*^ background [14], see Additional file 3: Figure S3A. Note that the arrows need to be reversed in Figure 5A so that it can be compared to the overexpressing *fz* clone, which is repulsive in character. If we take this clone to be *vang*-like, then the implication from our model is that Dsh is required for generating intrinsic polarity (model: *m*_*asym*_). If observed, adult patterns would most likely resemble Figure 5A close to the clone, though it is not possible to predict the pattern further out.

#### Polarity transmission absent (model: K^align^=0)

The second single *in-silico* background knockout corresponds to an absence of polarity transmission between the cells. A cell can still generate polarisation and align to a global signal. However, cells are not able to physically transmit their polarity to their neighbours. Figure 5B shows the resulting polarity pattern. It is fully polarised and aligned with the global field. However, the polarity is not influenced by the clone, appearing completely autonomous in character.

We found no matching pupal patterns in the literature, though similar observations have been found in the adult i.e. *fz* clones in *fmi-*[36], see Additional file 3: Figure S3B, and *dsh-* backgrounds [37]. The implication from adult wings is that Fmi and Dsh are required only to transmit cell polarity between cells (model: *K*^*align*^). This is a different inferred role for Dsh as compared to that in the pupal wing, which indicated it to be related to intrinsic cell polarisation.

#### Global polarity cue absent (model: C^mediator^ G^cue^=0)

The third single *in-silico* background knockout corresponds to either an absence of a global cue *G*^*cue*^ or of a mediator *C*^*mediator*^ that couples the cue to the local cell polarity. Polarisation can still be generated within a cell and cell-cell interactions are still able to convey polarity to neighbouring cells. Figure 5C shows the resulting polarity pattern. It is fully polarised, but complex in its appearance. Close to the clone, the pattern is radial in character as it is determined by the clone boundary shape, similar to that observed when intrinsic polarity is absent (model: *m*_*asym*_*=* 0). However, far from the clone there is near wildtype magnitude but nothing to influence the direction. This leads to a characteristic swirling patterns together with an interesting circular spiral (indicated by the arrow at the top of the figure) known in physics as a vortex singularity [32]. Traversing a path around the singularity would be represented by the angle θ changing through plus or minus 2π, dependent on the sign of vorticity.

Similar experimental observations are often described as having enhanced domineering non-autonomy. This enhancement is observed with *fz* clones in backgrounds lacking the activity of *ds* for both pre-hair patterns [22,35] and in the adult [38]. It is also seen with pre-hairs for *fz*+ clones (but with arrows reversed in Figure 5C) in backgrounds lacking the activity of Pk^pk-sple^[14], see Additional file 3: Figure S3C, or *ft*[22] and in adult hair backgrounds lacking the activity of Pk^pk^[39]. The implication from our model would be that both pre-hair and adult patterns indicate that Ds, Ft and Pk are required as part of the global signal or at least required to mediate it (model: *C*^*mediator*^*G*^*cue*^).

We now go on to consider more complex knockout situations.

#### Intrinsic polarity generation and transmission absent (model: m_asym_=0, K^align^=0)

**Figure 6:**
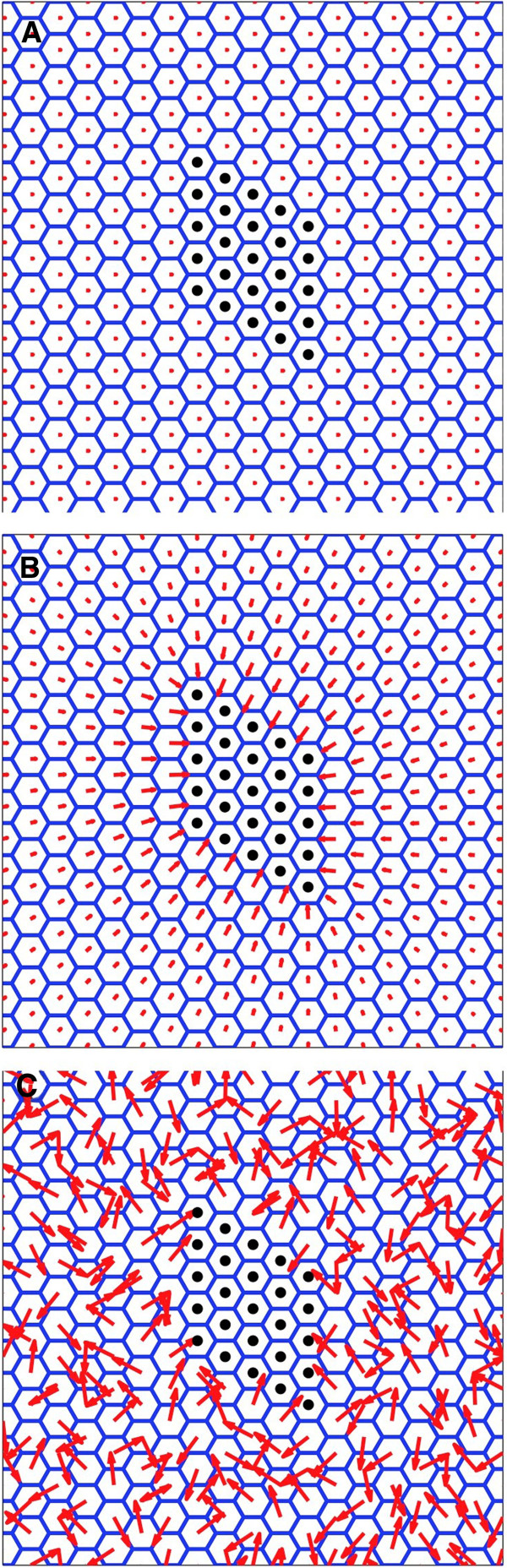
**Simulation of clones in a double mutant background.** Simulations of double knockouts using attractive (*fz*) clone simulations. (**A**) *m*_*asym*_*=* 0, *K*^*align*^*=* 0. (**B**) *m*_*asym*_*=* 0, *C*^*mediator*^***G***^*cue*^*=* 0. (**C**) *K*^*align*^*=* 0, *C*^*mediator*^***G***^*cue*^*=* 0. Repulsive (*Vang*) clones can be inferred directly by reversing the arrows of the attractive (*fz*) clones.

This double *in-silico* background knockout corresponds to the absence of molecular components that are necessary for establishing cellular polarisation and also those transmitting cell polarity to neighbouring cells. In Figure 5A we observed that the clone was able to transmit polarity to adjacent cells even in the absence of intrinsic polarity (*m*_*asym*_*=* 0). However in this case *K*^*align*^*=* 0 as well, which additionally removes influence by neighbouring cells, including cells in contact with the clone boundary. This means that the polarity can only be generated by an external signal. Figure 6A shows the resulting polarity pattern, which has negligible polarity everywhere, due to the external cue being insufficient to solely generate significant degree of polarisation.

Similar experimental patterns are observed in pupal wings with *fz* clones in a *Vang-* background and the reverse *Vang* clone in a *fz-* background [14,40], see Additional file 3: Figure S3D. Adult wings [11] for the same clone-background combinations look autonomous at short range (Figure 5B) and swirl at longer ranges (Figure 5C). No adult wings have been seen that resemble Figure 6A, most likely due to the inappropriate nature of our polarity indicators for adult hairs. The implication from our model would be that Fz and Vang are required for both the intrinsic generation and transmission of cell polarity using the pupal indicator and that they are required for transmitting polarity transmission and the global cue using an adult indicator.

#### Intrinsic polarity generation and global cue absent (model: m_asym_=0, C^mediator^ G^cue^=0)

This double *in-silico* background knockout corresponds to the absence of molecular components that are necessary for generating cellular polarisation and also those required for responding to a global cue. Naturally stable polarised cells are no longer possible, nor can they be influenced by global signals. Figure 6B shows the resulting polarity pattern. We observed some polarity, though it was only present close to the clone and transmitted equally in all directions. This pattern is similar to that observed in the single *m*_*asym*_ knockout, Figure 5A, though in that case the polarity was not equally transmitted along the proximal-distal axis.

The only comparable experimental observation that may match this phenotype is the polarity patterns observed in the pupal wing with a *fz*+ clone (but with arrows reversed in Figure 6B) in a *dsh-* background [14], see Additional file 3: Figure S3A, which led us to assign an intrinsic polarity generation role to Dsh. Therefore the implication from our model remains that Dsh is required for generating intrinsic polarity and that there exists a possibility that it may play a dual role in mediating the global cue (model: *m*_*asym*_ and *C*^*mediator*^*G*^*cue*^).

#### Polarity transmission and global cue absent (model: K^align^=0, C^mediator^ G^cue^=0)

This double *in-silico* background knockout corresponds to the absence of molecular components that are necessary for polarity transmission and also those required for responding to the global cue. Figure 6C shows the resulting polarity pattern. The polarity is well developed in each cell, but it is randomly orientated.

Interestingly, this pattern is not observed in any pre-hair or adult hair experiments.

Therefore the implication from our model is that no single protein included within our study has this dual role or that there may be redundancy present.

The relationship between these results and the phenotype descriptions is summarised in Table 1. Additional information relating to quantitative single and group order parameters, as described in Methods: Indicators of polarity can be found in supplementary Additional file 4: Table S1.

**Table 1:** Comparison of model and experimental phenotypes

**Genotype**		**Model function**				**Polarity phenotype**				** References**
**Clone**	**Background**	**m_asym_**	**K^align^**	**CG**	**Clone**	**Generic name**	**Figure**	**Adjacent to clone**	**Far from clone**	
none	WT				none	Wildtype		Proximodistal	Proximodistal	
*fz*	WT				Att	Attractive DNA	4A	Inward	Proximodistal	[8]
*vang*	WT				Rep	Repulsive DNA	4B	Outward	Proximodistal	[11]
*fj*	WT				Rep	Repulsive DNA	4B	Outward	Proximodistal	[43]
*fz+*	*dsh*	PH			Rep	Repulsive weak DNA	5A (Arrows reversed)	Outward	None	[14] (PH)
*fz*	*fmi*		H		Att/Rep	Autonomous	5B	Proximodistal	Proximodistal	[36] (H)
*fz*	*dsh*		H		Att/Rep	Autonomous	5B	Proximodistal	Proximodistal	[37] (H)
*fz*	*Vang*		H	H	Att	Autonomous	5B + 5C	Proximodistal	Swirling	[11] (H)
*Vang*	*fz*		H	H	Rep	Autonomous	5B + 5C	Proximodistal	Swirling	[11] (H)
*fz*	*ds*			PH/H	Att	Attractive enhanced DNA	5C	Inward	Swirling	[22,35] (PH)/[38] (H)
*fz*	*pk*^*pk*^			H	Att	Attractive enhanced DNA	5C	Inward	Swirling	[39] (H)
*fz+*	*pk*^*pk-sple*^			PH	Rep	Repulsive enhanced DNA	5C	Outward	Swirling	[14] (PH)
*fz+*	*ft*			PH	Rep	Repulsive enhanced DNA	5C	Outward	Swirling	[22] (PH)
*fz*	*Vang*	PH	PH		Att	No polarity	6A	None	None	[14] (PH)
*vang*	*fz*	PH	PH		Rep	No polarity	6B	None	None	[14] (PH)
*fz+*	*dsh*	PH		PH	Rep	Repulsive weak DNA	6B	Outward	None	[14] (PH)
			none	none		Random	6C	Random	Random	Not observed to date

Model function is either assumed to be wild type (left blank), knocked out as supported by adult hair (H) or pre-hair initiation (PH) observations. Domineering non-autonomous is abbreviated to DNA.

#### Polarity generation

Our simulations show that polarity is absent, except maybe close to a clone boundary, in all simulations that contained single or double mutant combinations involving *m*_*asym*_ =0. Therefore, we can eliminate any protein from a polarity generation role if we observe finite polarity away from a *fz* or *vang* clone.

We noted that polarity is observed in the pupal wing with *pk*^*pk-sple*^, *ft* and *ds* backgrounds [14,35]. The implication from our model would be that Pk, Ft and Ds proteins are at most weakly required for polarity generation, but not necessarily to determine polarity direction.

By contrast, we can assign polarity generation to any mutant combination in which pre-hairs emerge from the cell centre. This is clearly observed in *fz* and *dsh* backgrounds [41].

We are unable to repeat this process for adult wings as we cannot determine the magnitude of single cell polarisation.

#### Logical inferences from the model (clone boundary condition)

The final modification that can be made to our model is the application of the boundary condition at the clone boundary. Clones that lack a protein activity and appear like the classic *fz* (attractive) pattern can be placed within the proximal group, while those that appear like the classic *Vang* (repulsive) patterns can be placed into a distal group.

Experiments for both pupal and adult wings which exhibit attractive (classic *fz*) patterns include wildtype wings containing clones of only *fz*[8,14,35,40,42]. Experiments which exhibit repulsive (classic *Vang*) patterns include wildtype wings containing clones of Vang [11,14,40] and *fj*[43]. *ds*[22,35,38] clones also look attractive [39] and *ft* repulsive [22,44], however, they are weaker than their respective *fz* and Vang patterns.

The logical interpretation from our model is that the intrinsic proximal polarity group requires Vang and Fj and the distal group Fz. The weaker response seen in *ds* and *ft* clones indicates that they either weakly modify intrinsic polarity or locally reverse the global cue, as suggested for *fj*[43,45].

**Figure 7:**
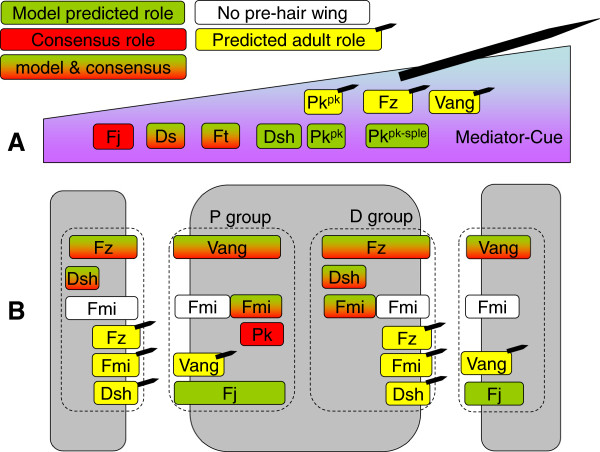
**Inferred and consensus functional roles of polarity proteins.** The top section (**A**) indicates proteins involved in a mediation or global cue role. We are unable to distinguish between the two using only our model. The lower section (**B**) indicates proteins with roles in polarity transmission or generation. Proteins located at the cell edge in the lower section are involved in transmitting polarity between cells (model: *K*^*align*^ ). Proteins located in the centre are involved in generating intrinsic polarisation (model: *m*_*asym*_ ). Proteins are also grouped into those required to generate cell proximal and distal ends (as seen in the wildtype). Green colouring indicates the roles predicted by the model and red indicates roles concluded form other evidence. Mixed red and green indicates that the model prediction is consistent with other evidence. Yellow with hair indicates a role predicted from adult hair data.

The functional protein roles predicted by our model together with logical inferences are summarised in Figure 7.

## Discussion

Mathematical modelling has long played a key role in developmental biology [46,47]. Models allow us to formalise our understanding of a given system and validate that formalisation by testing whether our knowledge is consistent with the modelling framework. Further, as increasingly large amounts of biological data accumulate, they allow us to manage our understanding to gain an overview of a system that may be too complex to hold in a head or lab notebook. Modelling approaches should allow experimental scientists to design improved experiments and approaches which will lead to a better understanding of the system under study. In this paper we describe the application of a simple modelling framework to a particular experimental system - the introduction of mutant clones into the developing *Drosophila* wing to analyse the genetic underpinnings of planar cell polarity. In doing this we organise and codify existing knowledge and, by showing that the results of our model are broadly consistent with the consensus view of the molecular functioning of the system, show that our modelling (conceptual) framework is appropriate for understanding the processes occurring during the development of PCP, at least at a high level. However our analysis also identifies potential novel roles for two proteins, Pk and Dsh, which we suggest may be mediators of the global cue. By making predictions of the results of defects in underlying processes we show that the experimental approach we model does not always allow unambiguous identification of the functions of genes, as different combinations of defects can give rise to similar patterns of hair polarisation. This should act as a driver for the development of new experimental approaches, and we have outlined below some quantitative measures that could improve understanding of the processes involved in PCP. A further advantage of the approach we have taken is that the theoretical patterns we predict for different combinations of defect may be used as direct indicators of the roles of mutated genes in the PCP process. This is not true for other kinds of models which model the detailed molecular interactions involved in PCP [25,28] and would need to be modified to characterise the roles of new components.

### Identification of molecular function

We generated six *in-silico* polarity patterns under different model conditions and compared them to *Drosophila* pre-hair and adult polarity patterns under different genetic conditions. Systematically knocking out model components has allowed us to annotate functional roles for proteins as defined by our model. We found that the “core” transmembrane proteins Fz and Vang are predicted to be required for simultaneously generating and transmitting polarity. This is almost certainly the case with Fmi, however, without pupal wings lacking the activity of Fmi-containing *fz* or *Vang* clones we could not confidently attribute such a role. These results are consistent with current consensus [21,23,24].

Ds and Ft were likely to be related to the global cue, though our model was unable to distinguish between a cue and/or mediation role. However, it looks increasingly likely that Ds and Ft are mediators with other experimental evidence arguing against a gradient role for Ft and Ds in the wing (although not the eye where they have been shown to alter ommatidial polarity) [48,49]. Weakly domineering non-autonomous patterns are observed with clones lacking the activity of *ds*[38] and *ft*[22,35,50]. This weak non-autonomy may at first glance appear to indicate a dual role in both “core” and “signal” roles. However, we do not think that this is the case for the following reason. A clone lacking a global signalling component would exhibit a step change in global signal strength (model: significant increase in G^*cue*^). Such a difference could be sufficient to influence the polarity at the clone boundary, giving a potential “core” like phenotype, though we have not confirmed this using our model.

A novel feature of this analysis is that we predicted more complicated roles for the “core” cytoplasmic proteins Pk and Dsh than their current consensus role of generating intrinsic polarity. We found that *pk* patterns compared more closely to our simulated cue knockout patterns than those involved in polarity generation. This may indicate the need for Pk’s role to be broadened and there is some recent evidence linking different isoforms of Pk to global patterning that support this [51]. We are less confident in our prediction for Dsh. This is due to the experimentally observed *dsh* patterns looking similar to both our simulated single intrinsic polarity knockout (Figure 5B, model: *m*_*asym*_*=* 0), a role in agreement with current consensus [21], and our simulated dual intrinsic and cue knockout pattern (Figure 6B, model: *m*_*asym*_*=* 0, CG *=* 0), indicating a new more complicated role for Dsh. Currently, we do not believe there are sufficient experimental observations to exclude an additional role for Dsh in mediating the global cue and that this novel role should be tested experimentally. A possible approach to more easily distinguish between these roles would be by generating long clones orientated along the anterior-posterior axis.

Randomly orientated polarity is expected when a protein has the dual role as a global cue or mediator (model: CG) and polarity transmission (model: *K*^*align*^). However, random orientation is not experimentally observed in our selected protein group or in any other genetic combinations within the literature. One possible explanation for this is that proteins involved in polarity transmission (model: *K*^*align*^) are always intrinsically related to polarity generation (model: *m*_*asym*_ ). This is consistent with the generation and transmission roles for Fz, Vang and most probably Fmi.

We focussed our attention to the polarity around clones. By doing so, we ensured that all terms in our model competed more or less equally together. Despite this, some of the polarity patterns we generated looked very similar to each other and were difficult to match to experimentally observed phenotypes, of which few were isolated and clear for comparison. This difficulty in making qualitative comparisons between experimental and modelling results presents challenges in functional annotation. We believe this would be best addressed by experimental approaches that provide quantitative polarity measurements such as those suggested in Methods (“Indicators of polarity”) and relate to our simulations in Additional file 4: Table S1. Of equal importance is focussed experimental design to create complex clone geometries where possible. Doing this would significantly aid an understanding of these spatially anisotropic processes. Away from the clones or in backgrounds absent of proteins, the three terms in our model do not necessarily compete equally and there could be different inferred functions, which we have not considered. This might even include areas of the wing where there is a reversal of polarity [38]. We could easily account for such a reversal in the model by allowing the mediator within the cue term to be spatially dependent in its sign.

Our model classified Ds and Ft as potential global signal mediators within the classic Fz and Vang pathway, and as Fj acts biochemically by modifying Ft and Ds binding [52,53], the non-autonomous phenotypes exhibited by *fj* clones are also likely to be mediated by effects on the global signal. Nevertheless, Fj, Ft and Ds also seem to be involved in the local coordination of polarity [19] and this could also contribute to their domineering non-autonomous phenotypes. Such a role of Ft and Ds in a parallel intersecting pathway to the Fz/Vang pathway is feasible, but cannot be directly tested in our current model. Interestingly, there is good evidence for such a parallel function in the *Drosophila* abdomen [17,18].

Pre-hair functional roles compared closely to the roles identified by our model for the adult hairs. This was near perfect when cellular polarisation was well established, though there were discrepancies when cellular polarisation was absent, in which case adult roles were assigned to polarity transmission and the global signal. The most likely explanation for the discrepancy is that our approach, in its current form, is not optimised for adult patterns. This is primarily because we are forced to assume only finite intrinsic single cell polarity for adult hairs. Magnitude information is more easily extracted from pre-hair patterns, which was our reason for focussing our attention to these phenotypes. Another explanation may be that the final adult polarity is guided, not by the fz-vang system, but by another mechanism or parallel polarity systems discussed earlier.

### Modelling

We based our deterministic model upon a single cell polarity measure, similar to [33,34]. This type of model is ideal for describing pre-hair initiation and adult hair orientation as it can often generate clear and reproducible phenotypic patterns. It does however, implicitly average stochastic effects within and between cells. We believe this to be a reasonable assumption as when others have included stochastic effects explicitly [29] they have yielded similar results to deterministic approaches. In contrast to these other models, our model explicitly identifies functional terms that are fundamental for coordinating long-range polarity. This is a powerful approach in that it does not pre-determine the role of any molecular component within the model whilst still providing a framework for molecular components to be assigned functional roles based on the observed polarity. Such an approach is also in contrast to models where the molecular interactions have been pre-assigned [25,28].

In order to parameterise our model, we compared the *in-silico* polarity patterns to those created by *fz* and *Vang* clones. We carried out this comparison in a qualitative manner paying attention to polarity in both a single cell and groups of cells. We found the parameter *K*^*align*^ to be at least the same magnitude as *B*^*asym*^. This means that on the cellular scale the overall strength of molecular interactions that act to align polarisation **inter**cellularly are of a similar magnitude as those that act to separate proximal and distal proteins **intra**cellularly. This similarity in strength may suggest that they have molecular components in common. While the equality is not necessarily a proof of common mechanism, it is consistent with the genetic evidence that Fz, Vang and Fmi are involved in both generating and transmitting polarity.

An additional constraint was that on a cellular scale the magnitude of the free energy required to maintain an individual cell’s polarisation *B*^*asym*^ is an order of magnitude greater than that needed to couple the cell polarity to a global cue, C^mediator^**G**^cue^. This makes biological sense in that it would be difficult to maintain global cues as strong as those that could be generated across a single cell. Finally, the magnitudes of the ratios of parameters were also not tightly constrained. Consequently the system should be robust with respect to a degree of biological noise which might be expected to vary over the spatial scale of the wing.

Our approach does have limitations. We chose to base our model and study on a relatively coarse polarity indicator i.e. the pre-hair initiation site rather than more detailed measures of protein distributions, which may provide a more precise indicator of polarity. We have also assumed a regular hexagonal grid and perfect exchange of polarity information. This is certainly not always the case [34,50], with some geometries likely to require a stronger global cue to counter the effects of irregular cell packing. How the cells sense polarity has also not been addressed here. We are using the model only to infer a molecule’s broad function and not the specific details of any particular interaction. We only compared the final equilibrium polarisation state and not the dynamical aspect of establishment of polarisation which is clearly important [26]. This was in part due to the absence of dynamical data required to identify the relevant time scales and processes for incorporation within the model. We have not explicitly considered partial levels of protein expression, though they are implicitly included through the ratios of parameters. The resulting behaviour is captured in summary form using Additional file 1: Figure S1 and Additional file 2: Figure S2, though the individual patterns are not shown.

We have presented only simulations of clones with regular shapes in this manuscript. We would expect, and preliminary data predict, that at large distances from the clone that shape makes practically no difference to patterns of polarity. However, at a closer distances the polarity may be significantly affected i.e. orientation and reduction in magnitude can be affected if the polarity field meets with a large clone wall perpendicular to the local polarity orientation. When meeting a wall at low angle the polarity should be maintained and the angle perturbed only slightly.

We presupposed the polarity on the clone boundary to remain unchanged from *fz* and *Vang* in wildtype for each mutant genetic background under study. This is equivalent to assuming that fz and Vang proteins are fundamental to polarity exchange between cells, currently the consensus.

This relatively simple model has enabled us to identify the majority of PCP protein functions using only 8 *in-silico* hair polarity patterns. This low number easily allows experimental researchers to formally assign high-level protein functions using experimental observations. Other more complicated models may include more specialised functions, but validation would be more difficult due to the number of genetic experiments that would be required. Interestingly, some of our results suggested that the model could be simplified further by combining the intrinsic polarity and transmission terms together. This would simplify the equation and implicitly link feedback loops to intrinsic polarisation involving adjoining cells. However, if we are to represent the feedback loops more directly it would require model development using more molecular-based approaches as has been considered elsewhere [28,29].

We did not make any assumptions about the nature of a global cue within our model, though we did identify molecular components that we believe to be related to it (Ds, Ft, Pk). These components have been linked to a competing polarity system (Ft-Ds-Fj) which may define the global cue. Other proposed mechanisms include hinge contraction and cell flow [20]. Each mechanism would generate a stress field within the wing that could be accounted for by our cue term (model: G^*cue*^ ). An attractive alternative is that it is the interaction between cells that induces cell flow, tissue elongation and polarity along the proximal-distal axis. Such a molecular interaction-induced flow is shown to occur in self organising liquid crystal phases [54]. Cell flow that is induced by cell-cell interactions is still likely to require an external morphogen cue or an anisotropic stress field to align polarity along the P-D axis. Therefore morphogen signals may still be essential for generating polarity on a global scale and also compatible with both stress and flow field theories.

## Conclusions

We built a functional model of cellular polarisation and used it to identify the role of proteins involved in generating cell polarity in the *Drosophila* wing. This model incorporates mathematical terms required for the asymmetric separation of proteins, the coupling of polarisation between cells and the coupling to a global cue, which we have validated against *fz* and *Vang* clone wing hair phenotypes.

Using *in-silico* knockdowns of combinations of each of the three model components, we have been able to systematically simulate polarity phenotypes which we compared to experimental clones’ patterns to predict protein function.

In agreement with the current consensus we predicted that Fz, Vang and Dsh are required for generating intrinsic cell polarity and that Fz and Vang are additionally required for transmitting cell polarity between cells. Ds and Ft were predicted to be related to the global cue, though we were unable to identify whether their precise role was that of a cue or a mediator to it.

Novel predictions from our model and approach are that the protein Pk and possibly Dsh are mediators of the global cue. This opens up the possibility that they could be involved in determining polarisation on a global scale.

Given the success of our approach we believe that it could be straightforwardly applied to investigate protein function in the eye and abdomen by matching the results of novel gene modification experiments to likely underlying mechanisms, and can therefore serve as a useful tool for future experimental analysis. The model can be extended to incorporate cell flow and anisotropically induced stress fields.

## Methods

Before creating models, we must first set out a formal description of polarity to allow us to describe polarity phenotypes. In order to achieve this, it will be necessary to introduce formal methods to quantify polarisation. These methods will allow us to attach metrics to the observed polarity phenotypes aiding in their classification.

### Indicators of polarity

In the *Drosophila* wing three primary indicators of polarity can be used.

The first is the asymmetric subcellular localisation of the core planar polarity proteins. In wildtype wings the proximal-distal axis of the plane of the epithelium [16] is normally indicated by a subset of proteins, including Fz which localises distally and a subset including Vang which localises proximally.

The second indicator of polarity is the site of initiation of the actin-rich trichome, which is directly related to the sites of localisation of the core proteins [40,41,55,56]. If the core proteins are fully asymmetrically localised, then hair initiation occurs at the cell edge where Fz is localised (normally the distal edge). This displacement of initiation from the centre can be used as an indicator of polarisation magnitude and direction. Cells lacking core protein function or with symmetric core protein localisation in the plane of the tissue show hair initiation in the cell centre.

The third indicator of polarity is the final orientation of the adult hair, whose orientation is intimately linked to the site of hair initiation [41]. We will use the last two indicators throughout this paper, though our focus has been more towards the pre-hair indicator.

All these indicators can be represented as a vector **m** with magnitude *m* (where *m* represents the strength of polarisation) and direction *θ*, for each cell. In the wildtype wing, this vector will align with its “head” pointing distally and its “tail” pointing proximally. We will find it convenient to describe proteins located proximally in the wildtype wing cells as “proximal proteins” and those located distally in wildtype wing cells as “distal proteins”. Hence cell polarisation can be measured by looking at **intra**cellular differences between proximal and distal protein distributions.

For a single wildtype hair cell finite polarisation is observed when *m* >*0* and the polarity vector may adopt any angle *θ* relative to the proximodistal, though most likely along it. For a proximal or distal protein null mutant, wing hair initiation should occur at the cell centre and so the magnitude *m = 0* and consequently the angle *θ* is not defined, see Figure 2. A feature of adult hairs is they nearly always indicate a single cell orientation, even in the case of polarity mutants where the hairs might simply lie flat. It is also difficult to assign a polarisation magnitude *m*, the distance from the cell centre for example, using published images. Therefore we must assume that the polarisation is always finite in adult hairs and use caution in inferring functions from adult patterns. This in turn reduces the inferential power of our approach based on adult hair indicators alone.

Now let us consider a group of cells. The simplest such group is the group of cells in cellular contact to each chosen reference cell, though it could in principle be an arbitrarily large group. The group’s orientational properties can be described mathematically using a vector order parameter **M**, containing the orientation of the average vector θ_A_ and its magnitude *M*. As with the single cell vector **m**, **M** is a vector with magnitude and direction but derived at a coarser scale as shown in Figure 2.

The vectors or vector order parameters we have discussed can be used to formally classify different aspects of the observed phenotypes. Here we will use both types of order parameter. The single-cell-perspective order parameter will be used to construct our model and both the single and group order parameters will be used to describe our results, presented in supplementary Additional file 4: Table S1. They are necessary to describe the symmetry properties of a single cell and a group of cells that possess vector order. An alternative way of describing polarity phenotypes when vector directions are equivalent, for example the equal binding of a protein to each end of a cell, is by using a tensor order parameter as described elsewhere [20].

### Model

Our functional model includes physically relevant terms that account for the cell’s ability to maintain its own intracellular polarisation, interact with the polarity of adjoining cells and interact with a global field. We have chosen to keep our model as general as possible by defining it in terms of cellular polarisation only. Cellular polarisation and the asymmetry of distributed proteins are therefore implicitly linked in this model. While this type of model is familiar to physicists, being used in the study of ferromagnetism [31] and other condensed matter systems [32], it may not be so familiar to the developmental biologist. Therefore we will take some time to explain the details of each model term, complemented by a biophysical interpretation using generalised proximal and distal proteins.

The approach is to construct an ‘effective free energy function’ that contains mathematical terms that relate the free energy to changes in the polarity only, and not the free energy of the whole cell system. Modelling the whole cell system would require the inclusion of factors, like the chemical potential (comprising ATP hydrolysis for example) which maintain the physiological state of the wing far from equilibrium, though the total free energy can still be reduced by an amount equal to the ‘effective free energy’ through changes in polarity.

The first mathematical term is to account for a cell’s ability to generate a stable polarisation magnitude *m = m*_*asym*._, which may be observed as the asymmetric distribution of proximal and distal proteins or the hair initiation, for example. The exact form of the first term is designed so that the polarisation is independent of external cues and polarity information from neighbouring cells, though we acknowledge that this might not be the case in practice. This independence may still lead to finite polarisation, though there will be no preference in the direction of polarisation. Mathematically, this means that this term will only be dependent on the magnitude of the polarisation *m* and not its direction θ. Thermodynamics requires that a free energy cost must be paid for by departures of the polarisation *m* from the stable value *m*_*asym*,_, irrespective of its value. This cost is taken into account by *B*_*asym*_, whose magnitude is determined predominantly by the effective temperature (with a value equal to or greater than the ambient temperature) or stochastic fluctuations in the system and not the details of the underlying molecular machinery, which determine the value of *m*_*asym*_. As the temperature or internal fluctuations grow it becomes easy for the system to depart from *m*_*asym*_, accounted for by a reduction in *B*_*asym*_. We do not expect it to change in the experiments presented here, though it might do in experiments involving many genetic factors.

An important point to note in our approach is that we have attached no precondition as to how the intrinsic polarisation *m*_*asym*_ is generated. It may be through **intra**cellular or **inter**cellular interactions alone or a combination of both. How might the cell polarisation relate to the actual underlying biological process? If we assume that spatial inhomogeneity in protein localisation is the initiator of symmetry breaking, which seems the most plausible mechanism, then we know that a feedback or amplification process must exist in order to drive the asymmetric localisation of “core” proteins (proximal and distal). This amplification process could entirely occur within a single isolated cell, see for example [57], or through interaction with a neighbouring cell. An interaction with neighbouring cells would not necessarily need to convey polarity information between cells to amplify polarisation, though if it did it would suggest that this mathematical term should be combined with the next term. Thermodynamically, the asymmetric localisation of proximal and distal proteins is due to a balance between intermolecular forces, which act to spatially separate the competing protein species (most likely through binding), and entropic forces, which act to homogenise the protein species. In the wildtype, intermolecular forces overcome the entropic ones leading to protein separation and finite polarisation *m*_*asym*_ > 0. In the absence of the correct molecular machinery, as we expect is the case of some mutants, there are insufficient intermolecular forces to overcome the entropic forces and so there will be little protein separation and no intrinsic polarisation, i.e. *m*_*asym*_ will vanish. We acknowledge that polarity in real cells is likely to be more complex than described, though they must still obey thermodynamic principles.

A simplified possible pathway schematic is shown in terms of proximal and distal proteins beneath term 1 in Figure 3.

The second mathematical term is to account for a cell’s tendency to align its polarisation with its neighbours. This term accounts for a free energy cost whenever the polarisation **m** of a cell is different from its neighbours. The magnitude of this free energy cost comes from the differences in polarisation vectors between an arbitrary cell and its nearest neighbours, 6 in a regular hexagonal array. An important feature of this model is that the cells with low polarisation vectors can be more easily distorted than those with a high degree of polarisation. As was the case with the previous term, the free energy cost will also be proportional to a constant *K*^*align*^, which is directly related to the inter-cellular physical interactions. For simplicity, we have assumed that the polarity information is perfectly and equally communicated between cells. By doing so, we have essentially averaged stochastic effects between cells to the continuum limit. We have also considered the cell packing to be perfectly hexagonal. This is not always realised in practice [50] and would require a reduction in the magnitude of *K*^*align*^ and the inclusion of cell-cell variations, a level of complexity beyond the scope of this paper.

What physically might be responsible for the resistance to changes in alignment from a cell’s neighbours? The most obvious cause, but not necessarily the only one, is ligand binding between cells [25,28]. In this case, ligand binding between cells would be maximised when a cell’s polarisation aligns with its neighbours, maximising intermolecular forces between cells. Departures from uniform alignment lead to a reduction in intermolecular binding and an increase in free energy cost.

The third and last mathematical term accounts for the cell’s tendency to align its polarity in the direction of an overlying global polarising cue, denoted by the vector ***G***^*cue*^. In this model, the alignment free energy is reduced when the polarisation vector **m** coincides with the vector ***G***^*cue*^. Again, the saving will also be proportional to a constant *C*^*mediator*^ mediating the strength of the interaction between a cell’s polarity and the global cue. We point out that by choosing such a term we have made no prior assumption that the global cue is required to generate polarisation (model term 1) though we appreciate that in practice it might be.

We can again convey meaning to this term using generalised proximal and distal proteins in relation to a morphogen gradient, though there could be other equally valid interpretations. In this case, the polarisation vector would attempt to align in the direction of increasing gradient, provided that there is differential signal-induced binding strength between proximal and distal proteins.

The total effective free energy (*F*_*total*_) is obtained by integrating over the tissue area (*d*^*2*^*r*), with the minimum determining the equilibrium polarity.

To help understand how the total free energy minimum relates to the observed cell polarity patterns, it is useful to consider a wildtype wing and one that contains genetically abnormal polarity, i.e. a “clone”. In the wildtype case, the total free energy is minimal when there is maximum polarity *m = m*_*asym*_ (term 1 *=* 0), the polarity in a cell aligns with its neighbours (term 2 *=* 0) and the polarisation aligns with the global cue (term 3 is maximal). By contrast, the presence of some “clones” creates a region where the polarisation is required to respond in a way that potentially conflicts with the global proximal-distal orientation. Such a conflict can be reconciled in two ways to produce a minimum-free energy state. The first is the re-orientation of the polarity between the two conflicting regions. This re-orientation would be accompanied by a free energy penalty for mis-alignment between neighbours (term 2 > 0) and the global field (term 3 < optimal). The second is the reduction of the polarity surrounding the clone *m ≠ m*_*asym*_. This would be associated with the price of introducing an asymmetric free energy cost (term 1 > 0).

The precise outcome depends mainly upon the clone boundary shape and how it affects polarity adjacent to it. In turn, the clone shape might also be affected by type of genetic manipulation or be coupled to the resulting polarity pattern, but this feedback is beyond the scope of the present paper. From a modelling point of view, we need to define what happens at this boundary. We allow two possibilities:

#### Attractive clone (cells lacking distal activity)

The orientation is defined inwardly perpendicular to the clone boundary, for each cell touching the clone boundary. This is expected to occur in clones lacking distal protein activity, which would appear differentially rich in proximal proteins;

#### Repulsive clone (cell lacking proximal activity)

The orientation is defined outwardly perpendicular to the clone boundary, for each cell touching the clone boundary. This is expected to occur in clones lacking proximal protein activity, which would appear differentially rich in distal proteins.

Cells away from the clone boundary are free to orientate their polarisation as if they were in an infinitely large region. The presence of the clone could affect the transmission of a global cue, though we have chosen not to include such a possibility in this first version of the model as it would require an additional level of complexity.

### Computations

Our model has been solved on a hexagonal array of cells (41 by 41), only partly shown in the figures. The polarity is obtained by minimising the total free energy (as given in Figure 3). To do this, we adopted a variational calculus approach that yielded two coupled Euler-Lagrange equations, see for example [31]. In this solution, the Laplacian for each vector component is calculated for reference cell by ∇^2^m (reference cell) =∑(surrounding 6 cells) – 6×(reference cell). The equations were further constrained by the application of boundary conditions. To enforce orientation at the clone boundary to be normal to the clone boundary we applied Dirichlet boundary conditions and to allow near free relaxation away from the clone we applied Neumann boundary conditions. The resulting equations were then solved using an explicit time dependent finite difference method [58] on a standard PC running Matlab until equilibrium. All simulations were started from an initial negligible random polarisation state. All repeated simulations led to the same final polarisation states as shown in the manuscript, except for the vortex singularity in Figure 5C. This suggests that the resulting polarisation states are likely to be global minima, except for Figure 5C where multiple stable states are expected.

## Competing interests

The authors declare that they have no competing interests.

## Authors’ contributions

LDH conceived and carried out the modelling studies and drafted the manuscript. JMH contributed to the design of the study and drafted the manuscript. Both authors read and approved the final manuscript.

## Supplementary Material

Additional file 1: Figure S1 Parameter exploration of angular penetration. Distance (in cells) over which the clone perturbs the hair orientation (greater than π/16) from wildtype (zero), measured vertically from the clone. Distance plotted as a function of ratios CG/B and K/B. Black square indicates the parameters using in the simulations. Click here for file

Additional file 2: Figure S2 Parameter exploration of polarisation magnitude penetration. Distance (in cells) over which the cell polarisation has a magnitude greater than 90% of wildtype, measured vertically from the clone. Distance plotted as a function of ratios CG/B and K/B. Black square indicates the parameters used in the simulations. Click here for file

Additional file 3: Figure S3 Experimentally observed polarity patterns. Images for the purposes of qualitative comparisons between the functional mutant background simulations and observed polarity patterns. A) *Fz* + in *Dsh* (reproduced with permission, Developmental Biology: Elsevier.com [14], doi:10.1016/j.ydbio.2006.09.026) compares to Figure 5A (arrows reversed), model *m*_*asym*_*=* 0 and also to Figure 6B (arrows reversed), *m*_*asym*_*=* 0, *C*^*mediator*^***G***^*cue*^*=* 0; B) *fz* in *fmi* (adult) (reproduced with permission [36], Development: dev.biologists.org) compares to Figure 5B, model *K*^*align*^*=* 0 ; C) *fz* + in *Pk*^*pk-sple*^ (reproduced with permission, Developmental Biology: Elsevier.com [14], doi:10.1016/j.ydbio.2006.09.026) compared to Figure 5C, model *C*^*mediator*^***G***^*cue*^*=* 0; D) Vang in *fz* (reproduced with permission, Development: dev.biologist.org [40], doi:10.1242/dev.025205) compared to Figure 6A, model *m*_*asym*_*=* 0, *K*^*align*^*=* 0. Click here for file

Additional file 4: Table S1 *In-silico* polarity phenotypes. Model knockouts are described in terms of single and multi-cell polarity measures. *m* and *M* are the single and multi-cell order magnitude respectively. θ and θ_A_ are the single and multi-cell angle and average angle respectively. Winding indicates the angle changes by 2π when tracing a path around the clone. Domineering non-autonomous is abbreviated to DNA. Click here for file
