# Supplementary figures and images for: Functional modelling of planar cell polarity: an approach for identifying molecular function
Source: BMC Dev Biol. 2013 May 14;13:20. doi: 10.1186/1471-213X-13-20 (PMC3662592; doi:10.1186/1471-213X-13-20)

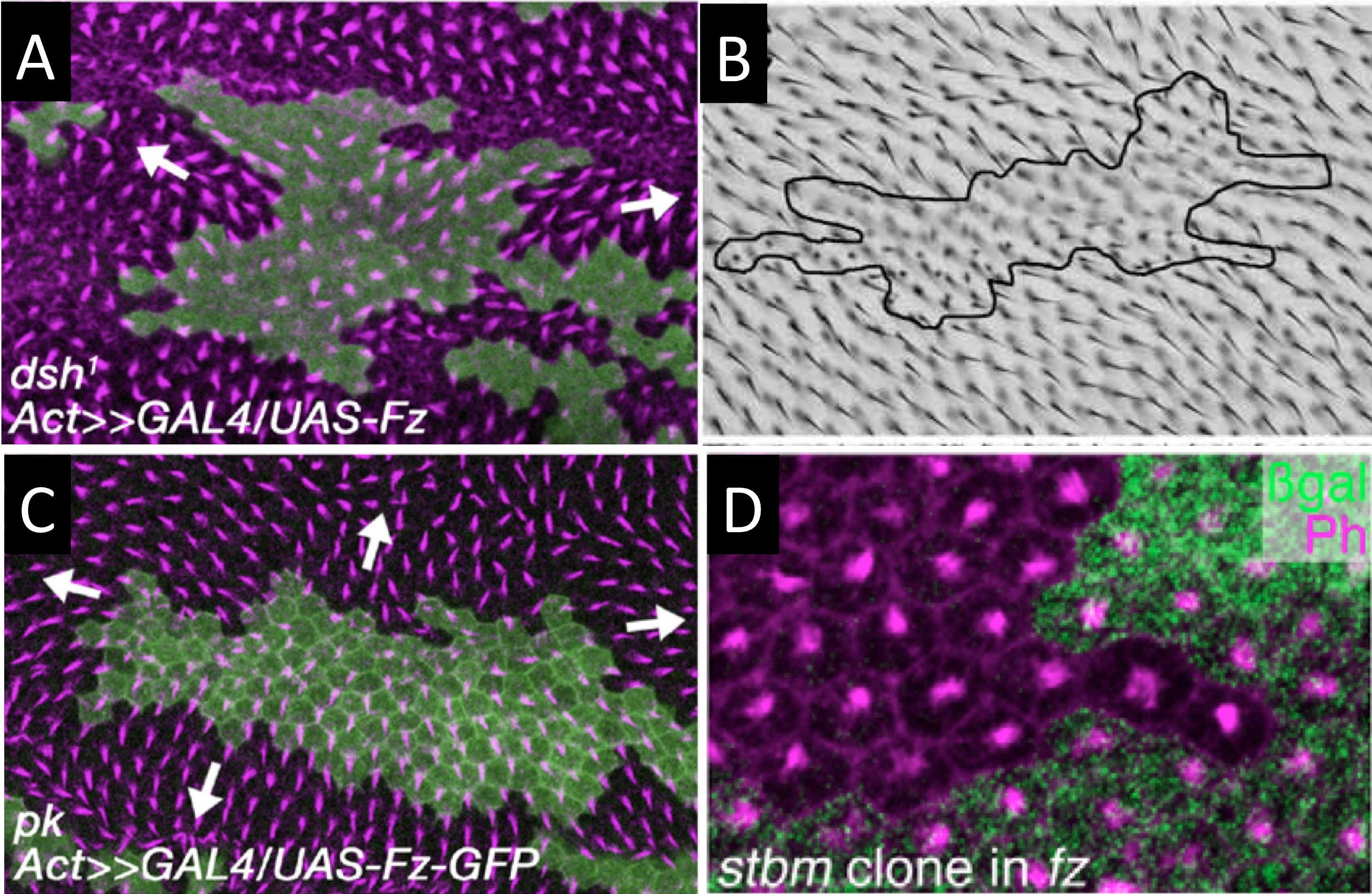

Supplement: Additional file 3: Figure S3 — Experimentally observed polarity patterns. Images for the purposes of qualitative comparisons between the functional mutant background simulations and observed polarity patterns. A) Fz + in Dsh (reproduced with permission, Developmental Biology: Elsevier.com [14], doi:10.1016/j.ydbio.2006.09.026) compares to Figure 5A (arrows reversed), model masym= 0 and also to Figure 6B (arrows reversed), masym= 0, CmediatorGcue= 0; B) fz in fmi (adult) (reproduced with permission [36], Development: dev.biologists.org) compares to Figure 5B, model Kalign= 0 ; C) fz + in Pkpk-sple (reproduced with permission, Developmental Biology: Elsevier.com [14], doi:10.1016/j.ydbio.2006.09.026) compared to Figure 5C, model CmediatorGcue= 0; D) Vang in fz (reproduced with permission, Development: dev.biologist.org [40], doi:10.1242/dev.025205) compared to Figure 6A, model masym= 0, Kalign= 0. [file 1471-213X-13-20-S3.jpeg]
