# Supplementary material for: Functional modelling of planar cell polarity: an approach for identifying molecular function
Source: BMC Dev Biol. 2013 May 14;13:20. doi: 10.1186/1471-213X-13-20 (PMC3662592; doi:10.1186/1471-213X-13-20)
Supplement: Additional file 4: Table S1 — In-silico polarity phenotypes. Model knockouts are described in terms of single and multi-cell polarity measures. m and M are the single and multi-cell order magnitude respectively. θ and θA are the single and multi-cell angle and average angle respectively. Winding indicates the angle changes by 2π when tracing a path around the clone. Domineering non-autonomous is abbreviated to DNA. [file 1471-213X-13-20-S4.doc]

Table S1. *In-silico* polarity phenotypes

Model knockouts are described in terms of single and multi-cell polarity measures. *m* and *M* are the single and multi-cell order magnitude respectively. θ and θ_A_ are the single and multi-cell angle and average angle respectively. Swirl is characterised by +2π or −2π change when tracing a path around the clone. Domineering non-autonomous is abbreviated to DNA.

| Model function | | | | Polarity Phenotype | | | | Polarity Measure | |
| --- | --- | --- | --- | --- | --- | --- | --- | --- | --- |
| m_asym_ | K^align^ | CG | Clone | Generic name | Figure | Adjacent to clone | Far from clone | Adjacent to clone | Far from clone |
|  |  |  | none | Wildtype |  |  | Proximodistal |  | m=m_asym_, θ=0  M=M _WT_, θ=0 |
|  |  |  | Att | Attractive DNA | 4A | Inward | Proximodistal | *m=m_asym_*, θ winding  M>0, θ_A_ winding | *m=m_asym_*, θ=0  M=M _WT_, θ_A_=0 |
|  |  |  | Rep | Repulsive DNA | 4B | Outward | Proximodistal | *m=m_asym_*, θ winding M>0, θ_A_ winding | *m=m_asym_*, θ=0  M=M _WT_, θ_A_=0 |
| KO |  |  | Att | Weak DNA | 5A | Inward | No polarity | *m<m_asym_*, θ winding M<M _WT_, θ_A_ winding | *m=0*  M=0 |
|  | KO |  | Att | Autonomous | 5B | Proximodistal | Proximodistal | *m=m_asym_*, θ=0  M=M _WT_, θ_A_=0 | *m=m_asym_*, θ=0  M=M _WT_, θ_A_=0 |
|  |  | KO | Att | Enhanced DNA | 5C | Inward | Swirling | *m=m_asym_*, θ winding  M=M _WT_, θ_A_ winding | *m=m_asym_*, θ=swirl  M=M _WT_, θ_A_=swirl |
| KO | KO |  | Att | No polarity | 6A | No polarity | No polarity | *m=0*  M=0 | *m=0*  M=0 |
| KO |  | KO | Att | Weak DNA | 6B | Inward | No polarity | *m<m_asym_*, θ winding  M<M _WT_, θ_A_ winding | *m=0*  M=0 |
|  | KO | KO | Att | Random | 6C | Random | Random | *m=m_asym_*, θ random M≈0 | *m=m_asym_*, θ random  M≈0 |
